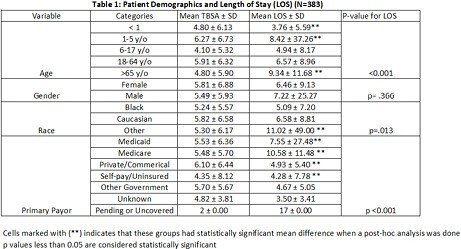# 737 Demographic Features and Length of Stay of Scald Injuries: Review from a Northeastern Burn Center

**DOI:** 10.1093/jbcr/irae036.280

**Published:** 2024-04-17

**Authors:** Vivian Chung, Joan Dolinak, Tamara L Roberts, Angela T Wratney

**Affiliations:** SUNY Upstate Medical University, Syracuse, NY; Upstate University Hospital, Not Hispanic or Latino, NY; Suny Upstate Medical-Clark Burn Center, Syracuse, NY; SUNY Upstate Medical University, Manlius, NY; SUNY Upstate Medical University, Syracuse, NY; Upstate University Hospital, Not Hispanic or Latino, NY; Suny Upstate Medical-Clark Burn Center, Syracuse, NY; SUNY Upstate Medical University, Manlius, NY; SUNY Upstate Medical University, Syracuse, NY; Upstate University Hospital, Not Hispanic or Latino, NY; Suny Upstate Medical-Clark Burn Center, Syracuse, NY; SUNY Upstate Medical University, Manlius, NY; SUNY Upstate Medical University, Syracuse, NY; Upstate University Hospital, Not Hispanic or Latino, NY; Suny Upstate Medical-Clark Burn Center, Syracuse, NY; SUNY Upstate Medical University, Manlius, NY

## Abstract

**Introduction:**

Multitude of demographic and clinical features that affect the incidence and outcome of a burn injury have been identified. However, prognostic factors associated with the incidence and length of stay (LOS) of scald specific injuries is still unclear. The present study aims to identify and understand the factors that affect LOS in patients with scald injuries that present to a Northeastern tertiary burn center and characterize populations at risk for scald injury.

**Methods:**

A retrospective nonexperimental descriptive study was performed from the record of all burn patients presenting to our ABA verified burn center from January 01, 2015 to December 31, 2022. Data of patients with scald injuries were isolated and organized using an Excel Spreadsheet. Scald admission variables reviewed and analyzed included: age, sex, race, suspected abuse, TBSA, Surgical visits, payor source, and LOS. SPSS version 29.0 was used to obtain descriptive statistics and perform Kruskal-Wallis 1-Way ANOVA and Mann Whitney U. Statistical significance was defined at p< 0.05

**Results:**

A total of 2266 patients were admitted to the burn center during 2015-2022. From this, 383 patients were admitted for scald burns, which is 16.9% of all burn admissions. Scald burn patient’s demographics included male (62.1%), female (37.9%) with a mean age of 26.82. Major race was Caucasian (62.4%) and the primary payor source was Medicaid (52.7%). Approximately 12% of these cases were identified as suspected abuse. Of these abuse cases, pediatric patients composed the majority (80.4%). There was no significant difference in LOS when patients were stratified by sex (p=.366) or whether suspected abuse was involved (p=.186). However, there were differences in distribution of LOS when patients were stratified by age (p < .001), race (p=.013), surgical visits (p < .001), and primary payor (p < .001) The mean TBSA was 5.6%. There was no significant difference in the distribution of TBSA when patients were stratified by age, abuse, sex, race, or payor source. However, the association between TBSA and total surgical visits was found to be significant.

**Conclusions:**

Longer LOS was associated with patients > 65 when compared to patients < 18 and with patients 18-64 when compared to patients 5 and under. Longer LOS was also associated with Medicare patients in comparison to Medicaid, Private/Commercial, and self pay patients. The association between Race and LOS was also found to be statistically significant when comparing Caucasians to patients in the “Other” group. This study highlighted differences in scald injuries between age, race, and primary payor method.

**Applicability of Research to Practice:**

The risk factors for scald specific injuries differ from previous literature addressing wider burn etiologies. Identification of populations at increased risk for scald injuries can guide public health initiatives and prevention.